# Effects of a Proprietary Standardized *Orthosiphon stamineus* Ethanolic Leaf Extract on Enhancing Memory in Sprague Dawley Rats Possibly via Blockade of Adenosine A_**2A**_ Receptors

**DOI:** 10.1155/2015/375837

**Published:** 2015-11-08

**Authors:** Annie George, Sasikala Chinnappan, Yogendra Choudhary, Vandana Kotak Choudhary, Praveen Bommu, Hoi Jin Wong

**Affiliations:** ^1^Biotropics Malaysia Berhad, Lot 21 Jalan U1/19, Section U1, Hicom-Glenmarie Industrial Park, 40150 Shah Alam, Selangor, Malaysia; ^2^Ethix Pharma Laboratories, Karbala Road, Bilaspur 495001, Chhattisgarh, India

## Abstract

The aim of the study was to explore a propriety standardized ethanolic extract from leaves of* Orthosiphon stamineus *Benth in improving impairments in short-term social memory* in vivo,* possibly via blockade of adenosine A_2A_ receptors (A2AR). The ethanolic extract of* O. stamineus *leaves showed significant* in vitro *binding activity of A2AR with 74% inhibition at 150 *μ*g/ml and significant A2AR antagonist activity with 98% inhibition at 300 *μ*g/mL. A significant adenosine A_1_ receptor (A1R) antagonist activity with 100% inhibition was observed at 300 *μ*g/mL. Its effect on learning and memory was assessed via social recognition task using Sprague Dawley rats whereby the ethanolic extract of* O. stamineus *showed significant (*p* < 0.001) change in recognition index (RI) at 300 mg/kg and 600 mg/kg p.o and 120 mg/kg i.p., respectively, compared to the vehicle control. In comparison, the ethanolic extract of* Polygonum minus *aerial parts showed small change in inflexion; however, it remained insignificant in RI at 200 mg/kg p.o. Our findings suggest that the ethanolic extract of* O. stamineus *leaves improves memory by reversing age-related deficits in short-term social memory and the possible involvement of adenosine A_1_ and adenosine A_2A_ as a target bioactivity site in the restoration of memory.

## 1. Background

*Orthosiphon stamineus *Benth (Lamiaceae) is a herbaceous perennial plant, widely distributed throughout the tropical regions, especially in Southeast Asia. It is commonly known as cat's whiskers. It is also known as misai kuching in Malaysia and kumis kuching in Indonesia [1]. It is referred to as java tea and consumed as an herbal tea in Europe for urinary flushing (*European Herbal Pharmacopoeia*). The leaves of* O. stamineus* are traditionally used in South East Asia for a variety of ailments such as bladder and kidney disease (due to its strong diuretic effect), detoxification, relieving joint stiffness and inflammation including arthritis and rheumatism, gout, treating catarrh of the bladder, eliminating stones from the bladder, and treating diabetes mellitus [2, 3]. Scientific studies have further reported the herb to possess anti-inflammatory [4], antioxidant [5, 6], antibacterial [7], hepatoprotective [8], diuretic [9], antihypertensive [10], and hypoglycemic effects [11].

Several classes of bioactive compounds such as flavonoids, diterpenes, triterpenes, saponins, sterols organic acids, caffeic acids derivatives, chromenes, and oleanic and ursolic acid are known for* O. stamineus* [12–16]. Recent studies have emerged on the flavonoids of* O. stamineus* possessing antagonist activity on adenosine A_1_ receptors (A1R) [17]. While the study focused more on the role of the receptors in diuretic activity, adenosine receptors in the central nervous system have also been implicated in the modulation of cognitive functions [18]. While the A1R antagonist activity has been reported in* O. stamineus*, A2AR antagonist activity was not.

The adenosine receptors have been associated with sleep and arousal, cognition, and memory and with protecting from neuronal damage and degeneration as well as influencing neuronal maturation [19]. Endogenous adenosine is generally known to modulate cognition through the activation of adenosine A_1_ receptors. Evidence is now emerging on a possible role of A_2A_ receptors in learning and memory [20]. The adenosine receptors A_1_ and A_2A_ belong to the G-protein-coupled receptor family [18] and antagonist actions on these receptors produced CNS-enhancing effects. Selective blockade of A_1_ and A_2A_ receptors were shown to facilitate learning and memory* in vivo *[21, 22]. They might also protect against memory dysfunction shown in experimental models of aging such as Alzheimer's disease.

The social recognition test (SRT) has been used in studies with caffeine, an adenosine A_2A_ receptor antagonist, in reversing cognitive decline in age-related deficits in olfactory discrimination, Parkinson's disease, and attention deficit hyperactivity disorder (ADHD) [21, 23]. Using the social recognition test, an adenosine A_2A_ receptor antagonist demonstrated the ability to reverse short-term memory loss in Spontaneously Hypertensive Rats (SHR) which have impairments across several cognitive domains such as attention, short-term memory, and spatial reference memory [20].

The social recognition test was first introduced by Thor and Holloway [24] and is based on the premise that rodents spend more time with unfamiliar juveniles than familiar ones. Memory-enhancing drugs are used in this model to investigate whether the duration of investigation is reduced when the juvenile rat is presented twice. The social recognition test in rats has become increasingly popular for the pharmaceutical industry as a tool to evaluate compounds for procognitive activity. This memory test probes short-term recognition/working memory to investigate novel target mechanisms relevant to cognitive impairment including neuropsychiatric disorders such as dementia, Alzheimer's disease (AD), schizophrenia, and Parkinson's disease (PD). Importantly the test uses spontaneous naturalistic behavior of an adult rat when exposed to a juvenile conspecific on two occasions to access cognition, where the output measured (recognition index (RI)/ratio of investigation duration between the two sessions) involves an assessment of social exploration, strongly influenced by an olfactory component. As a result, SRT animal model was selected in this study.

Antagonists to A2AR are not the only target when seeking cognition enhancing treatment. The inhibitory effects on other target sites such as acetylcholinesterase and serotonin have shown improvement in memory and cognition. One such plant preparation shown to possess antiacetylcholinesterase activity, a neurotransmitter related to learning and memory, is the standardized extract of* Ginkgo biloba*. Standardised extracts of* G. biloba* were shown to improve memory and normalized cognitive deficits in animal models [25, 26]. Meanwhile, leaves of another Malaysian herb,* Polygonum hydropiper,* have been reported to also possess antiacetylcholinesterase activity and recently its related species* P. minus* demonstrated enhanced memory in rats study using the Barnes maze test and demonstrated anticholinesterase activity [27]. The purpose of this study is to evaluate* O. stamineus *leaf ethanolic extract for cognition-improving benefits and adenosine A_2A_ receptor as a possible target. The effect is compared with* G. biloba* and* P. minus *extract, for memory improvement in an SRT animal model.

## 2. Materials and Methods

### 2.1. Extract and Drug

#### 2.1.1. *O. stamineus *Leaves

*O. stamineus *leaves, of white flower variety, procured from Biotropics Malaysia Berhad, Malaysia, were harvested at maturity approximately 3 months after planting. The plant material was identified on the basis of exomorphic characters and literature review by a taxonomist from the Institute of Bioscience, Universiti Putra Malaysia (UPM). The voucher specimen of* O. stamineus *(SK 2083/12) was deposited in the Herbarium, Institute of Bioscience, UPM of Malaysia.

#### 2.1.2. Ethanolic Extract of* O. stamineus *Leaves

1000 g of* O. stamineus *leaves was dried by oven at a temperature of 40°C for 48 hours and ground into a fine powder using a lab mill (Retsch ZM200, Haan, Germany) and was extracted twice with 2 L and 1.5 L of 70% ethanol in water (v/v) using ultrasonic treatment for a period of 30 min at room temperature. The solution was separated from the remaining material. The organic solvent was removed under reduced pressure at 40°C and dried.

#### 2.1.3. HPLC Analysis of* O. stamineus* Ethanolic Extract

The extract was characterized using HPLC techniques based on seven known compounds of* O. stamineus* used as reference standards [28]. The compounds were 3′-hydroxy-4′,5,6,7-tetramethoxyflavone, sinensetin, orthosiphol B, orthosiphol A, staminol A, orthosiphonone A, and ombuin (3,3′,5-trihydroxy-4′,7-dimethoxyflavone). HPLC analysis of the extract was performed using Agilent 1200 Liquid Chromatography (LC) with a photodiode array detector on Zorbax Eclipse XDB-C18, 4.6 × 150 mm, 5 *μ*m column. The mobile phase consisted of solvent A: water and solvent B: acetonitrile. The following gradient was used: 0–8 min, 70% A; 8–15 min, 70–53% A; 15–30 min, 53–49% A, hold for 10 min; 40–42 min, 49–0% A, hold for 4 min; 46–48 min, 0% A for final washing and equilibrium of the column for the next run. Operating conditions were set at flow rates of 1 mL/min, column temperature at 25°C, UV detection at 230 nm, and injection volume of 5 uL. The extract at the concentration of 50 mg/mL was first injected followed by the mixture of the standards. Identification of the marker compounds was achieved by comparing with retention times of reference standards and their UV spectra.

#### 2.1.4. Aqueous Extract of* P. minus*

1000 g of aerial parts including stem and leaves of the plant was harvested at maturity approximately 2 months after planting and was dried by oven drying at the temperature of 40°C for 48 hours and shredded to 2 to 5 cm in size. The dried leaves were extracted according to the method described in George et al. [27]. The dried leaves were then subjected to percolation using purified water and extracted at a temperature of about 80°C with an extraction ratio of approximately 1 : 10. The extract was further filtered, concentrated using rotary evaporator with the water bath temperature of 65°C, and freeze-dried. The voucher specimen of the plant (SK 2077/12) was deposited in the Herbarium, Institute of Bioscience, UPM, Malaysia.

### 2.2. *In Vitro* Adenosine Receptors A_2A_ and A_1_ Assays

The adenosine A_2A_ receptor (A2AR) and A_1_ receptor (A1R) assays were performed to determine test item's A2AR and A1R blockade activity.* O. stamineus* extract was tested at 15 and 150 *μ*g/mL for A_2A_ binding assay, and the method employed was adapted from the one described by Varani et al. [29]. Adenosine A_2A_ and adenosine A_1_ functional assays were performed at 3,30 and 300 *μ*g/mL and the method was adapted from Paucher et al. [30] and Taylor et al. [31], respectively. Adenosine A_2A_ binding assay, selective adenosine A_2A_, and adenosine A_1_ antagonist assays were conducted by Eurofin Panlabs (previously known as Ricerca) with test catalog numbers of 200610, 300500, and 401000, respectively. Reference standards were run as an integral part of all three assays to ensure the validity of the results. The assays were performed under conditions described in Tables 1 [Table tab2]–3.

### 2.3. Animals

Ninety adult male SD rats (3-month-old, 200–250 g) and juvenile male rats of the same strain (35–40-day-old, 75–100 g) from the National Institute of Nutrition, Tarnaka, Hyderabad, were used as described in Table 4. The diet comprised standard pellet diet by Provimi (Nutrilab Rodent). Juvenile rats were kept in groups of ten per cage and served as social stimuli for the adult rats. The animals were maintained in a room under controlled temperature (22 ± 2°C), with relative humidity of between 50 and 70% and were subjected to a 12 h light cycle (lights on 8:00 a.m.) with free access to food and water. All the experimental procedures (IAEC/CPCSEA approval number 1412/a/11 in February 2012) were performed according to the guidelines on animal care of the OECD Principles of Good Laboratory Practice, as revised in 1997 and adopted on November 26th, 1997, by decision of the OECD Council [C(97)186/Final].

### 2.4. Treatment

The plant extract of* O. stamineus* (doses 60, 120, 200, 300, and 600 mg/kg b.w.), a commercial extract of* G. biloba* (120 mg/kg, standardised to 27.25% Ginkgo flavonglycosides, 6% Terpene lactones, and ≤ 5 ppm ginkgolic acid determined through HPLC methods), water extract of* P. minus *(200 mg/kg), and the drug donepezil (ARICEPT tablet, Zydus Cadila Ltd., 3 mg/kg) were dissolved in distilled water. The control solution consisted of distilled water (vehicle). The extract of* O. stamineus* was tested i.p. and orally. Extracts of* O. stamineus* at doses of 60 and 120 mg/kg b.w. and donepezil at 3 mg/kg b.w. were administered i.p. for a direct comparison to donepezil activity, 120 min before the second encounter C2. In addition, extracts of* O. stamineus* at doses of 200, 300, and 600 mg/kg b.w.,* G. biloba* extract at a dose of 120 mg/kg, a concentration derived from past animal studies of* G. biloba* in cognition-related investigations [32], and 200 mg/kg water extract of* P. minus* (as a direct comparison with the lower dose of the test extract) and vehicle were administered orally, 120 min before the second encounter C2.

### 2.5. Social Recognition Test

Short-term social memory was assessed with the SRT described by Mondadori et al. [33]. Nine groups of rats, each consisting of 10 males, were used for the study. Adult Sprague Dawley (SD) rats were housed individually in polycarbonate cages and they were used only after at least 7 days of habituation to their new environment. The test was scored in a consistent manner in an observation room, where the rats had been habituated for at least 1 h before the beginning of the test. All juveniles were isolated in individual cages for 30 min prior to the beginning of the experiment. The SRT consisted of two successive presentations (5–10 min each) separated by a short period of time where a juvenile rat was placed in the home cage of the adult rat and the time (s) spent by the adult in investigating the juvenile (nosing, sniffing, grooming, or pawing) was recorded (C1). At the end of the first presentation, the juvenile was removed and kept in an individual cage during the delay period and reexposed to the adult rat after 120 min and time (s) spent by the adult in investigating the juvenile was recorded (C2). In this paradigm, a reduction in the investigation time during the second encounter reflects the recognition ability of the adult rat. A pretest was performed for verification that the test compounds themselves do not have effects on social investigation per se. In this experiment, a different juvenile to the one used in the first presentation was exposed to the adult rat during the second encounter, with a similar duration of social investigation time being expected. RI was calculated using the formula (RI = C2/C1) for social recognition assay.

All values are expressed as means ± SEM (*n* equals the number of rats included in each analysis). The RI (RI = C2/C1) was calculated for social recognition assay. The data was analyzed by comparing control versus treatment and standard and changes in activity before and after treatment (C1 versus C2) and RI versus control, standard, and treatment using Student's *t*-test by Graph Pad Prism 4.0 software.

## 3. Result

### 3.1. Characterization of* O. stamineus* Ethanolic Extract

**Figure 1:**
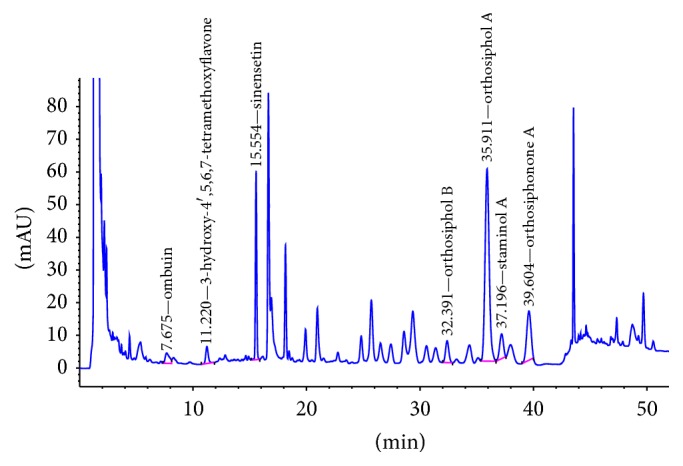
HPLC chromatograms of* O. stamineus* leaf ethanolic extract.

**Figure 2:**
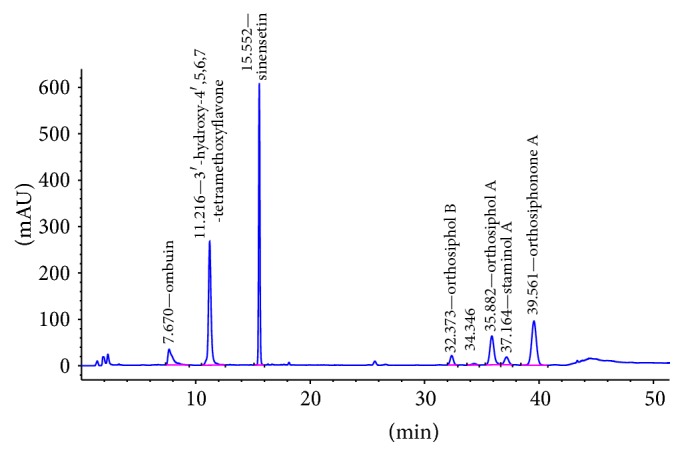
HPLC chromatograms of reference standard compounds. The identified peaks are ombuin (3,3′,5-trihydroxy-4′,7-dimethoxyflavone), 3′-hydroxy-4′,5,6,7-tetramethoxyflavone, sinensetin, orthosiphol B, orthosiphol A, staminol A, and orthosiphonone A.

Chromatographic profile of* O. stamineus *ethanolic extract composition and reference compounds are as shown in Figures 1 and 2, respectively. The peaks corresponding to selected seven compounds were identified based on retention time against reference standards, and the UV spectrum. The peaks of ombuin (3,3′,5-trihydroxy-4′,7-dimethoxyflavone) (0.14%), 3′-hydroxy-4′,5,6,7-tetramethoxyflavone (0.10%), sinensetin (0.07%), orthosiphol B (0.26%), orthosiphol A (0.67%), staminol A (0.45%), and orthosiphonone A (0.12%) are eluted at retention times 7.675 min, 11.220 min, 15.554 min, 32.391 min, 35.911 min, 37.196 min, and 39.604 min, respectively. The resulting standardized extract is based on the group of marker compounds.

### 3.2. *In Vitro *Adenosine A_2A_ Receptor (A2AR) and Adenosinse A_1_ Receptor (A1R) Assays

**Figure 3:**
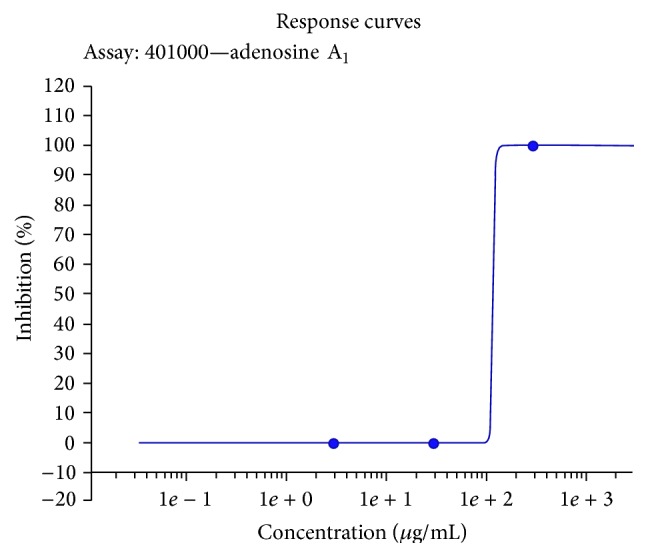
Response curve for adenosine A_1_ antagonist assay. ^*∗*^The IC_50_ of adenosine A_1_ antagonist assay for* O. stamineus* ethanolic extract is 95.1 *μ*g/mL.

**Figure 4:**
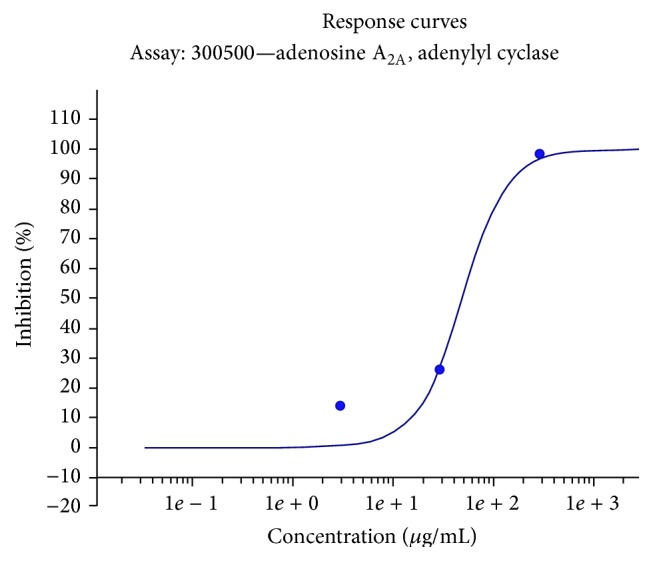
Response curve for adenosine A_2A_ antagonist assay. ^*∗*^The IC_50_ of adenosine A_2A_ antagonist assay for* O. stamineus* ethanolic extract is 51.5 *μ*g/mL.

The ethanolic extract of* O. stamineus *leaves showed significant binding activity with 74% inhibition of A2AR at a dose of 150 *μ*g/mL and antagonist activity in the A_2A_ functional assay at 300 *μ*g/mL with 98% inhibition of cAMP response induced by NECA (Table 5). The extract showed similar activity in A1R inhibition, with an antagonist activity at 300 *μ*g/mL where the extract displayed 100% inhibition of response induced by cyclohexyladenosine (CHA). The antagonist activity of the* O. stamineus *leaves ethanolic extract to adenosine A_2A_ and adenosine A_1_ receptors suggests the biological activity of* O. stamineus* in an* in vitro *system. The IC_50_ for A2AR binding activity is estimated at 60.07 *μ*g/mL and determined with nonlinear regression analysis by Inplot GraphPad Prism, San Diego, CA, computer program. The IC_50_ for A1R antagonist is 95.1 *μ*g/mL (Figure 3). The IC_50_ for A2AR antagonist based on the response curve is 51.5 *μ*g/mL (Figure 4). The Ki value for the A2AR binding assay is calculated using the Cheng-Prusoff equation (1973) and is estimated at 33.72 mM.

### 3.3. Social Recognition Test

In the SRT procedure, SD rats presented a clear impairment of the juvenile recognition ability (recognition index) in comparison to control rats (*p* < 0.001), since control group spent as much time investigating the juvenile rat during the second encounter as they did on the first exposure. The difference between treated and control groups on juvenile recognition ability is showed with more details in Table 6, with detailed analysis of the investigation time. The investigatory behaviour of the adult SD rats was concentrated in the first 5 min of the juvenile presentation, with a significant reduction in the investigation time during the second encounter 120 min later. The effects of the administration of acute doses of* O. stamineus* extract (200, 300, and 600 mg/kg, p.o., and 60, 120 mg/kg, i.p.),* P. minus* (200 mg/kg, p.o.),* G. biloba* (120 mg/kg, p.o.), donezepil (3 mg/kg, i.p.), and the vehicle (p.o.) in the SD rats social investigation time are given in Table 6.* O. stamineus* extract has shown significant (*p* < 0.001) change in RI compared to vehicle control at an oral dose of 300 mg/kg and 600 mg/kg, respectively. It also exerted significant (*p* < 0.001) change in RI at a dose of 120 mg/kg i.p. compared to vehicle control. However, 200 mg/kg oral and 60 mg/kg i.p. dose remained insignificant for* O. stamineus *extract. The reduction in inflexion was further confirmed with significant (*p* < 0.05, *p* < 0.001, and *p* < 0.05) change in activity before (C1) and after (C2) treatment for* O. stamineus* extract group, at oral doses of 300 mg/kg, 600 mg/kg, and 120 mg/kg i.p., respectively (Table 4, C1 versus C2 significance). The extract of* P. minus* and* G. biloba *has shown small change in inflexion; however it remained insignificant for RI compared to vehicle, at an oral dose of 200 mg/kg and 120 mg/kg. The standard drug donepezil dosed at 3 mg/kg i.p. has shown change in inflexion but no significant change in RI as compared to vehicle control.

## 4. Discussion

The chemical constituents and the A2AR binding activity of* O. stamineus *extract have demonstrated that, with a single treatment of* O. stamineus* leaves extract after C1, the time spent in scrutinizing the same partner at a second meeting, 120 min later, is shortened. The extract-induced reduction of the exploration time can be attributed to learning of the specific information of the partner retained from the first meeting that reduced the need for new information. The assumption that specific attributes of a particular partner were remembered is strengthened by the significant RI (*p* < 0.001) seen with the test extract. The ethanolic extract of* O. stamineus *leaves showed significant activity with 74% inhibition of A2AR at a dose of 150 *μ*g/mL. Therefore, the study suggests the possible binding of the* O. stamineus *extract to A2AR, attributing the social recognition task with this biological activity.

The present results demonstrate that the SD rats present a significant impairment of short-term social memory in SRT in the vehicle group as the RI of more than 1 signifies no improvement in recognition (RI should be <1). In fact, a longer time to recognize juvenile rat (C2) was observed for the vehicle group. The findings also suggest the involvement of the adenosine receptors in this response, since the acute administration of* O. stamineus *leaves extract reversed this social memory deficit in SD rats. Several studies have demonstrated that the selective blockade of adenosine A_1_ and adenosine A_2A_ receptors facilitates learning and memory in rodents models [22, 34]. The Ki value of the extract in this experiment was 33.72 mM. Caffeine and theophylline, another naturally occurring xanthine mainly found in tea, are nonselective AR antagonists. Their stimulating properties are associated with micromolar range affinities for the A2AR. Although caffeine and theophylline have similar* in vitro* affinities for the A_2A_ receptor, caffeine has a higher stimulating effect due to a higher brain unbound fraction wt a Ki value of 23400 nM [35]. Though caffeine derivatives possess stronger A2AR binding activities, the multicompounds that exist in* Orthosiphon stamineus* extract including flavonoids may have affected memory more than one way possibly also via other receptors such as the inhibition of acetylcholinesterase, thereby enhancing cognition [36]. Furthermore, the extract tested in this study also possessed additional A1R antagonist activity, as previously reported in* O. stamineus* [17]. From the SRT, promnesic property of* O. stamineus *leaves extract was observed in SD rats at dose dependent manner of 300 mg/kg and 600 mg/kg p.o. and 60 mg/kg and 120 mg/kg i.p. This study revealed that the* O. stamineus* leaves extract (300 and 600 mg/kg, p.o.; 60 and 120 mg/kg i.p.) exerted significant activity when compared to standard donepezil (3 mg/kg i.p.) statistically reaching significance in all except at 60 mg/kg i.p. where RI was only almost significant (*p* < 0.0539). The* O. stamineus* leaves extract appears to prevent the amnesic effect of the long delay (120 minutes) where such a preventive effect may be deduced as promnesic. The extract at 120 mg/kg i.p. was comparable to 300 mg/kg and 600 mg/kg orally dosed in RI, signifying greater bioavailability when administered i.p. at only one-third of the oral dosage.

As for* G. biloba* extract, a 120 mg/kg oral dose failed to demonstrate significant activity in this study but in another study, a single i.p. injection of* G. biloba* extract at 120 mg/kg dose demonstrated improvement in recognition performances in young rats in a similar olfactory animal model study [32]. This may be due to* G. biloba* having a lower bioavailability when administered through nonintravenous route (as observed in this study). The herb* P. minus* on the other hand has been shown to possess antiacetylcholinesterase activity in a recent study [27] although in the current study improvement in recognition index was not significant. The promnesic effects are probably more apparent in a model that tests attention rather than learning and memory from olfactory cues. This is in parallel to findings by Blokland [37] where it was suggested that the role of acetylcholine in learning and memory processes was still not conclusive, rendering its role more important in attention processes. A different animal model such as Barnes maze that tests spatial learning and memory instead of memory by olfactory and social cues, such as in this study, may have been a better model to investigate* P. minus* [27].

Based on the mean of RI for the extracts given i.p., donepezil fared better (in terms of lower mean) than the plant extract of* G. biloba* at their tested dosage though the route of administration was different. A2AR activity has never been known for* G. biloba* though anticholinesterase activity has been reported [25] and* in vivo* memory improvement has been documented for* G. biloba* extracts [26]. Donepezil, a reversible inhibitor of cholinesterase which is clinically used for treatment of dementia, showed slightly weak (RI at *p* < 0.0593) cognition enhancing properties at 3 mg/kg i.p. The effect of 3 mg/kg donepezil is similar to 60 mg/kg i.p. of* O. stamineus* extract based on the mean at C1 and having changes to RI after treatment at *p* < 0.0593 and *p* < 0.0539, respectively. This may be due to it being a popular acetylcholinesterase inhibitor for memory impairment which is age-related and long term such as Alzheimer and dementia. The short reaction time (120 minutes) and the use of adult but not aged rats to impart a significant effect in the RI may have contributed to the weaker response in the Donepezil group.

Neurodegenerative disease can be the result of neuronal cell death caused by oxidative stress, apoptosis, and inflammation. Apart from A2AR activity, alcohol extracts of* O. stamineus *leaves may possess other biological activities that are neuroprotective. They have been reported to possess antiapoptotic effects in a H_2_O_2_ (a potent free radical) induced cell apoptosis [5]. The antioxidant properties of* O. stamineus *in addition may play a positive role in the prevention of neurogeneration caused by damaging free radicals [7]. The* O. stamineus* is known to contain several classes of bioactive compounds such as flavonoids, diterpenes, triterpenes, saponins, sterols organic acids, caffeic acids derivatives, chromenes, and oleanic and ursolic acid, known for [12–16]. Flavonoids have been shown to possess antioxidative and anti-inflammatory effects that suggest neuroprotective property [36]. Oleanic acid which has been isolated from* O. stamineus* has been reported to protect against neuronal death induced by beta-amyloid in cultured rat cortical neurons and improve beta-amyloid induced memory deficit in mice [38]. Ursolic acid reduced the production of proinflammatory cytokines and neurotoxic reactive oxygen species, thus possibly leading to an additional neuroprotective effect [39].

Caffeine is another example of adenosine A_2A_ receptor antagonist that modulates the release of different neurotransmitters in the olfactory bulb of rodents [40] known to play a role in social olfactory recognition [41]. It appears to be that* O. stamineus *extract behaves similarly to caffeine in improving short-term memory and alertness.* O. stamineus*, however, does not contain caffeine but is rich in terpenoids and flavonoids. Terpenoids from natural products such as* G. biloba* and Asian ginseng (*Panax ginseng*) are currently being investigated as potential therapeutics in Alzheimer's disease, already showing some promise [42]. Terpenoids were identified in the* O. stamineus* extract used, that is, orthosiphol B and orthosiphol F, staminol A, and orthosiphonone A, which makes this extract a potential candidate for further investigation in the area of cognition disorder.

## 5. Conclusion

Our findings suggest that the propriety standardized ethanolic extract of* O. stamineus* may reverse age-related deficits in short-term social memory and can be considered to prevent or decrease the rate of neurodegeneration. The further investigation of not only adenosinergic but also other neurotransmitters in producing improvements in cognition should be evaluated in the future. The involvement of A_1_ and A_2A_ blockade in the social memory deficit can be further clarified in their role in the* Orthosiphon stamineus* effects along with selective A_1_ and A_2A_ antagonist assayed in a social recognition tests for confirmation of target.

## Figures and Tables

**Table 1:** Adenosine receptor A_2A_ binding assay parameters.

Adenosine A_2A_	
Source	Human recombinant HEK-293 cells
Ligand	0.05 *μ*M [3] CGS-21680
Vehicle	1% DMSO
Incubation time/temp.	90 minutes at 25°C
Incubation buffer	50 mM Tris-HCl, pH 7.4, 10 mM MgCl_2_, 1 mM EDTA, 2 U/mL adenosine Deaminase
Nonspecific ligand	50 *μ*M NECA (5-N-ethylcarboxamide adenosine)
KD	0.064 *μ*M
*B*_max⁡_	7 pmole/mg protein
Specific binding	85%
Quantitation method	Radioligand binding
Significance criteria	≥50% of max stimulation
Reference	CGS-21680

**Table 2 tab2:** Adenosine receptor A_2A_ functional assay parameters.

Adenosine A_2A_ adenylyl cyclase	
Source	Human recombinant HEK-293 cells
Control	0.1 *µ*M NECA
Vehicle	0.40% DMSO
Incubation time/temp.	10 minutes at 37°C
Incubation buffer	Modified Hank's balanced salt solution (HBSS) pH 7.4
Quantitation method	HTRF quantitation of cAMP accumulation
Significant criteria for agonist	≥50% increase in cAMP relative to NECA response
Significant criteria for antagonist	≥50% inhibition of NECA-induced cAMP increase

**Table 3:** Adenosine receptor A_1_ functional assay parameter.

Adenosine A_1_	
Source	Wistar rat vas deferens
Control	0.3 *μ*M CHA (N6-cyclohexyladenosine)
Vehicle	0.10% DMSO
Incubation time/temp.	5 minutes at 32°C
Incubation buffer	KREBS pH 7.4
Quantitation method	Isometric (gram changes)
Significant criteria for agonist	≥50% reduction of neurogenic twitch relative to 0.3 *μ*M CHA response
Significant criteria for antagonist	≥50% inhibition of 0.3 *μ*M CHA-induced relaxation

**Table 4:** Animal grouping according to test materials, dose, and route of administration.

Group number	Dose level	Route	Number of animals
1	Vehicle control	p.o.	10
2	BT 00119 (200 mg/kg)	p.o.	10
3	BT 00119 (300 mg/kg)	p.o.	10
4	BT 00119 (600 mg/kg)	p.o.	10
5	PME 00012 (200 mg/kg)	p.o.	10
6	GBE 000120 (120 mg/kg)	p.o.	10
7	Donepezil (3 mg/kg)	i.p.	10
8	BT 00119 (60 mg/kg)	i.p.	10
9	BT 00119 (120 mg/kg)	i.p.	10

**Table 5:** Results of* in vitro* adenosine A_2A_ and adenosine A_1_ assays.

Assay	Concentration (*μ*g/mL)	Inhibition (%)	IC_50_ (*μ*g/mL)
Adenosine A_2A_ binding assay	15	17	60.07
	150	74	

Adenosine A_2A_ functional assay antagonist	3	14	51.5
	30	26	
	300	98	

		Increase in cAMP (%)	
Adenosine A_2A_ functional assay agonist	3	−1	—
	30	−3	
	300	−8	

Adenosine A_1_ functional assay antagonist activity	3	0	95.1
	30	0	
	300	100	

		Reduction in neurogenic twitch (%)	
Adenosine A_1_ functional assay agonist activity	3	5	—
	30	12	
	300	29	

**Table 6:** Effect of *O. stamineus* (BT 00119), *P. minus *(PM 00012), *G. biloba *(GBE 00110), and donepezil on recognition index with respect to duration of interactions in social recognition test in the SD rats.

Treatment (mg/kg) p.o./i.p., immediately after C1		Investigation duration (seconds)				C2 versus C1	Recognition index (C2/C1)	
	Route	First contact (C1)		Second contact (C2) 120 min after C1				
		Mean ± SEM	*p* value	Mean ± SEM	*p* value	*p* value	Mean ± SEM	*p* value
Vehicle	p.o.	22.00 ± 11.59	—	25.00 ± 13.0	—	0.1777	1.107 ± 0.20	—
BT 00119 (200)	p.o.	28.67 ± 5.36	0.3146	14.33 ± 8.51	0.2656	0.1273	0.5033 ± 0.24	0.0661
BT 00119 (300)	p.o.	57.33 ± 7.53^a*∗*^	0.0315	14.33 ± 2.90	0.2348	0.0119^b*∗*^	0.2400 ± 0.02	0.0068^c*∗∗∗*^
BT 00119 (600)	p.o.	71.00 ± 7.81^a*∗*^	0.0124	8.333 ± 1.85	0.1374	0.0051^b*∗∗∗*^	0.1133 ± 0.017	0.0042^c*∗∗∗*^
PME 00012 (200)	p.o.	48.67 ± 6.88	0.0595	31.00 ± 5.68	0.3475	0.1419	0.6833 ± 0.18	0.0986
GBE 00110 (120)	p.o.	52.67 ± 5.69^a*∗*^	0.0382	33.67 ± 2.84	0.2759	0.0775	0.6600 ± 0.11	0.0651
Donepezil (3)	i.p.	30.67 ± 1.20	0.2492	18.33 ± 4.33	0.3266	0.0621	0.5500 ± 0.11	0.0593
BT 00119 (60)	i.p.	26.67 ± 4.80	0.3644	8.667 ± 5.23	0.1550	0.1801	0.3867 ± 0.28	0.0539
BT 00119 (120)	i.p.	51.00 ± 2.51^a*∗*^	0.0354	14.00 ± 2.51	0.2272	0.0136^b*∗*^	0.2733 ± 0.05	0.0086^c*∗∗∗*^

p.o. = per oral, i.p. = intraperitoneal, and SEM = standard error mean.

^a*∗*^ *p* < 0.05 indicates the significance of first contact in comparison with vehicle control for all groups.

^b*∗*^ *p* < 0.05 and ^b*∗∗∗*^ *p* < 0.001 indicate the significance in comparing the change in activity before and after treatment.

^c*∗∗*^ *p* < 0.01 and ^c*∗∗∗*^ *p* < 0.001 indicate the significant changes of RI when compared with vehicle control.
